# The acid-tolerant L-arabinose isomerase from the mesophilic *Shewanella *sp. ANA-3 is highly active at low temperatures

**DOI:** 10.1186/1475-2859-10-96

**Published:** 2011-11-10

**Authors:** Moez Rhimi, Goran Bajic, Rimeh Ilhammami, Samira Boudebbouze, Emmanuelle Maguin, Richard Haser, Nushin Aghajari

**Affiliations:** 1 Laboratory for BioCrystallography and Structural Biology of Therapeutic Targets, "Bases Moléculaires et Structurales des Systèmes Infectieux" UMR5086 CNRS/Université de Lyon1, Institut de Biologie et Chimie des Protéines FR3302, 7 Passage du Vercors, F-69367 Lyon cedex 07, France; 2 Institut National de la Recherche Agronomique, UMR 1319 Micalis, F-78350 Jouy-en-Josas, France

**Keywords:** L-arabinose isomerase, acid-tolerant, cold-active, D-tagatose

## Abstract

**Background:**

L-arabinose isomerases catalyse the isomerization of L-arabinose into L-ribulose at insight biological systems. At industrial scale of this enzyme is used for the bioconversion of D-galactose into D-tagatose which has many applications in pharmaceutical and agro-food industries. The isomerization reaction is thermodynamically equilibrated, and therefore the bioconversion rates is shifted towards tagatose when the temperature is increased. Moreover, to prevent secondary reactions it will be of interest to operate at low pH. The profitability of this D-tagatose production process is mainly related to the use of lactose as cheaper raw material. In many dairy products it will be interesting to produce D-tagatose during storage. This requires an efficient L-arabinose isomerase acting at low temperature and pH values.

**Results:**

The gene encoding the L-arabinose isomerase from *Shewanella *sp. ANA-3 was cloned and overexpressed in *Escherichia coli*. The purified protein has a tetrameric arrangement composed by four identical 55 kDa subunits. The biochemical characterization of this enzyme showed that it was distinguishable by its maximal activity at low temperatures comprised between 15-35°C. Interestingly, this biocatalyst preserves more than 85% of its activity in a broad range of temperatures from 4.0 to 45°C. *Shewanella *sp. ANA-3 L-arabinose isomerase was also optimally active at pH 5.5-6.5 and maintained over 80% of its activity at large pH values from 4.0 to 8.5. Furthermore, this enzyme exhibited a weak requirement for metallic ions for its activity evaluated at 0.6 mM Mn^2+^. Stability studies showed that this protein is highly stable mainly at low temperature and pH values. Remarkably, T268K mutation clearly enhances the enzyme stability at low pH values. Use of this L-arabinose isomerase for D-tagatose production allows the achievement of attractive bioconversion rates of 16% at 4°C and 34% at 35°C.

**Conclusions:**

Here we reported the purification and the biochemical characterization of the novel *Shewanella *sp. ANA-3 L-arabinose isomerase. Determination of the biochemical properties demonstrated that this enzyme was highly active at low temperatures. The generated T268K mutant displays an increase of the enzyme stability essentially at low pH. These features seem to be very attractive for the bioconversion of D-galactose into D-tagatose at low temperature which is very interesting from industrial point of view.

## Background

L-Arabinose isomerase, hereafter L-AI, (EC 5.3.1.4) converts L-arabinose into L-ribulose within living systems [1]. This enzyme is also known as D-galactose isomerase due to its capacity to isomerize D-galactose into D-tagatose *in vitro *[2,3]. Over the past years, rare sugars have made their way to the market and the demands have been increased as in agro-food industry rare natural sugars such as D-tagatose have become of a great interest as sweeteners [4]. Indeed this natural sugar has the taste and natural properties of sucrose but it is an anti-hyperglycemiant factor known to reduce symptoms associated with type 2 diabetes [5,6]. D-Tagatose has not only a very low caloric value of 1.5 kcal/g (only 30% of the energy content of sucrose), but has also been reported to promote weight loss [5]. All these properties are very interesting for industrials, yet the use of this sugar is quite limited due to its cost [7]. In fact, chemical methods used at present to produce D-tagatose are very expensive. Therefore, biological methods using L-arabinose isomerase as a catalyst are being developed due to its low cost [8].

Many micro-organisms such as *Arthrobacter, Lactobacillus, Mycobacterium, Klebsiella *and *Gluconobacter *are able to convert D-galactitol into D-tagatose [9]. However, this production procedure is not attractive due to the high cost of the raw material D-galactitol. That is why studies are now investigating a new bioprocess, which consists in converting D-galactose into D-tagatose using L-AIs. These studies have shown that the reaction equilibrium shifts towards D-tagatose production with increased temperatures, and that the majority of L-AIs are thermoactive biocatalysts requiring metal ions for their maximal thermostability and activity [3,10-14]. Unfortunately, for industrial purposes high temperatures and alkaline pH result in the formation of side reactions generating undesirable by-products that should be removed (together with the metal ions) at the end of the process, thus increasing the production costs [7,15]. For these reasons, highly thermoactive L-AIs deriving from thermophilic strains such as *Bacillus stearothermophilus *US100, *Thermotoga maritima, Thermotoga neapolitana, Geobacillus thermodenitrificans *and *Thermus *sp. were identified [12,13,16-18]. Many industrial applications including bioconversion of D-galactose to D-tagatose during products storage, which must be done at low temperatures, require an L-AI that acts not only at acidic pH but also at low temperatures. To satisfy these requirements, screening of novel L-AIs has been done. As a result, many L-AIs were identified and characterized to be functional at relatively high pH and at high temperatures [10-18]. Lately, four L-AIs from *Alicyclobacillus acidocaldarius, Bacillus licheniformis*, *Lactobacillus plantarum *NC8 and *Lactobacillus sakei *23 K strains were described to be acid-tolerant. Molecular determinants for the catalytic efficiency were explored by homology modeling followed by site-directed mutagenesis [19,20]. Protein engineering was also used to generate enzymes acting at low temperatures, such as the N175H mutant from *B. stearothermophilus *US100 [15,20-22] and at acidic pH (mutant Q268K) [22]. On the basis of these two mutants a third one was developed (Q268K/N175K) [22]. This double mutant is highly active at both acidic pH and low temperatures making it very promising for industrial D-tagatose production. Nevertheless, it remains of interest to set out a stable L-AI active at even lower temperatures.

Here we report the cloning, over-expression, purification and biochemical characterization of an L-AI isolated from *Shewanella *sp. ANA-3. The activity and stability of the enzyme at low temperatures is discussed in comparison with previously described L-AIs. Site-directed mutagenesis has been performed to generate the T268K mutant and explore its biochemical properties.

## Results and discussion

### Gene, cloning and over-expression

**Figure 1:**
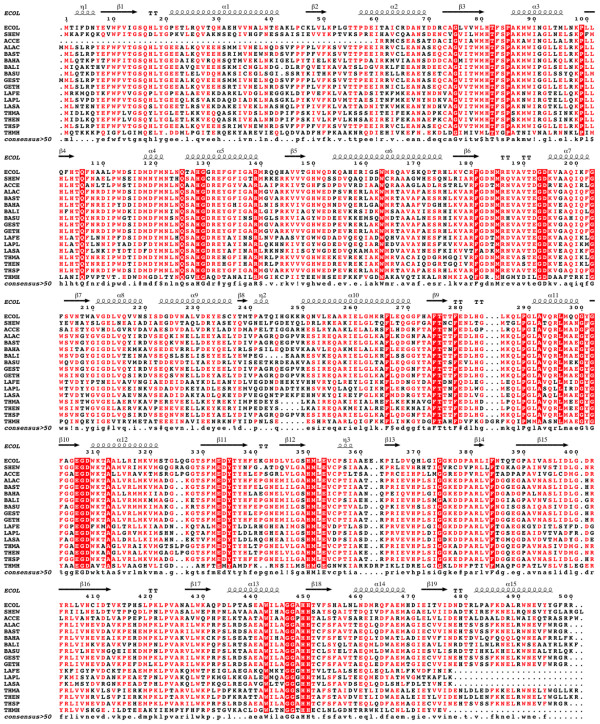
**Multiple sequence alignment**. Invariant residues between sequences are typed red on a white background and residues conserved within each group are displayed as white letters on a red background. ECOL, *E. coli *(accession number PO8202); SHEW, *Shewanella *sp. ANA-3 (accession number A0KWX7); ACCE, *A. cellulolytics *ATCC 43068 (accession number GU188440); ALAC., *Alicyclobacillus acidocaldarius *(accession number AAY68209); BAST, *B. stearothermophilus *US100 (accession number CAI29261); BAHA, *B. halodurans *(accession number NP_242739); BALI, *Bacillus licheniformis *(accession number YP_080173.1); BASU, *B. subtilis *(accession number ACT82395); GEST, *G. stearothermophilus *(accession number ABY84698); GETH, *G. thermodenitrificans *(accession number Q5QKM7); LAFE *L. fermentum *(accession number ADJ94948); LAPL, *L. plantarum *(accession number Q88S84); LASA, *Lactobacillus sakei *23 K (accession number YP_396468.1); THMA, *T. maritim*a (accession number Q9WYB3); THEN, *T. neapolitana *(accession number Q8RMB9); THSP, *Thermus *sp. (accession number AY225311); THMH *T. mathranii *(accession number ADH61392).

A DNA fragment of approximately 1.5 kb was obtained by polymerase chain reaction (PCR) amplification using *Shewanella *sp. ANA-3 chromosomal DNA as template and the two oligonucleotides. DNA was ligated into the pGEMT-Easy vector and then transferred into the *Escherichia coli *BL21 (DE3) host strain. Numerous white colonies were observed and subsequently analyzed with the appropriate restriction endonucleases; a recombinant clone carrying the *araA *gene under the control of T7 promoter was obtained (pMR37). Then, the *araA *gene was sub-cloned in pET-15b under control of the T7 promoter leading to the pMR38 plasmid. Monitoring of liquid culture (BL21/pMR38 strain), followed by an L-AI activity test in the crude extract, showed an activity of 26 U/mg, confirming the molecular cloning and the expression of *Shewanella *sp. ANA-3 *araA *under the control of the T7 promoter. This latter gene encodes a 500 amino-acid residues protein with a calculated molecular mass of 55.3 kDa. Inspection of the amino-acid sequence multiple alignment of this protein with several other L-AIs indicated that *Shewanella *sp. ANA-3 L-AI displays the highest sequence identity (54.5%) with L-AI from the acidophilic strain *A. acidocaldarius*. Other enzymes such as those from *B. licheniformis, L. plantarum *NC8 and *L. sakei *23 K, share 50.5%, 40.7% and 38.7% sequence identity, with *Shewanella *sp. ANA-3 L-AI, respectively (Figure 1). Sequence alignments with the thermophilic *G. stearothermophilus, B. stearothermophilus *US100, *B. thermodenitrificans*, *T. neapolitana *and *T. maritima *showed significative identity of 54.2%, 54.4%, 54.3%, 47.7% and 48.3%, respectively (Figure 1).

### Purification of the *Shewanella *sp. ANA-3 L-AI

**Figure 2:**
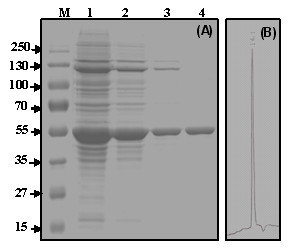
**Electrophoretic and size exclusion FPLC analysis of the purified *Shewanella *enzyme**. (A) SDS-PAGE showing the various steps in the purification procedure under reducing conditions. Lane M, protein markers (molecular masses in kDa); lane 1, total cell extract; lane 2, sample from lane 1 after ammonium sulphate precipitation; lane 3, sample from lane 2 after affinity chromatography; lane 4, purified recombinant L-AI after size exclusion chromatography. (B) Size exclusion HPLC analysis of the purified recombinant *Shewanella *sp. ANA-3 L-AI shows a single peak of 230 kDa (RT = 14.1 min), using protein markers of 669 kDa (RT = 8.755 min), 440 (RT = 13.165 min), 232 (RT = 14.02 min; RT, retention time), 140 (RT = 17.163 min) and 66 (RT = 21.438 min).

After an over-night liquid cell culture of the BL21/pMR38 strain, the protein crude extract was subjected to fractioned precipitation with ammonium sulfate (Figure 2A). The subsequent step consisted in loading the resolubilized fractions onto a HiTrap™Chelating Immobilized Metal Affinity Chromatography (IMAC) column. A high purity enzyme extract was achieved with size exclusion chromatography (Figure 2A). The single elution peak obtained has an apparent molecular mass of 230 kDa (Figure 2B). Electrophoresis under reducing conditions (12% SDS-PAGE; sodium dodecyl sulfate-polyacrylamide gel electrophoresis) revealed a homogenous band with a molecular mass of about 55 kDa (Figure 2A), suggesting that *Shewanella *sp. ANA-3 L-AI is a tetramer composed by four monomers of 55-kDa. Previous studies showed that all L-AIs have a similar, homotetrameric quaternary structure [11,13-15,18] the exception being *E. coli *L-AI which forms a homohexamer [23]. Presently, the only known L-AI 3D-structure is that of *E. coli *[23], and further structural studies would be of high interest in order to explore the importance of the quaternary structures in D-galactose binding and/or catalysis. *Shewanella *sp. ANA-3L-AI presented a relatively high specific activity of 164 U/mg making it more active than most L-AIs from mesophilic bacterial origin (Table 1). Only few of the currently characterized L-AIs exhibit higher specific activity, mainly those from thermophilic bacteria like *B. stearothermophilus *US100 (185 U/mg) and *T. neapolitana *(119 U/mg) or lactic acid bacteria as *L. sakei *(218 U/mg) [10,13,24].

**Table 1:** Biochemical properties of previously reported L-AIs.

Organism	Topt (°C)	pH	Metal ion Requirement (mM)	*K*m (mM)	*V*max (mmole/s/mg)	References
*Shewanella *sp. ANA-3	15-35	5.5-6.5	Mn^2+ ^(0.6)	33.7*	164*	This study
*Acidothermus cellulolytics*	75	7.5	Mn^2+ ^(1.0), Co^2+ ^(0.5)	NR	NR	[35]
*Alicyclobacter acidocaldarius*	50	6.0	Mn^2+ ^(1.0), Co^2+^, (1.0) Mg^2+ ^(1.0)	48	35.5	[15]
*Anoxybacillus flavithermus*	95	9.5-10.5	Ni^2+ ^(1.0)	78.5	94.3	[25]
*Bacillus stearothermophilus *US100	80	7.5-8.0	Mn^2+ ^(1.0), Co^2+ ^(0.2)	28.57	185	[13]
*Bacillus halodurans*	50	7.5-8.0	Mn^2+ ^(1.0)	36	33.1	[27]
*Bacillus licheniformis*	50	7.5	Mn^2+ ^(1.0), Co^2+ ^(1.0)	369	NR	[14]
*Bacillus subtilis*	32	7.5	Mn^2+ ^(1.0)	120	NR	[36]
*Escherichia coli*	30	8.0	Mn^2+^(0.5)	60	NR	[37]
*Geobacillus stearothermophilus *T6	70	7.0-7.5	Mn^2+ ^(1.0)	63	36.5	[1]
*Geobacillus thermodenitrificans*	60	7.5	Mn^2+ ^(5.0), Co^2+ ^(3.0)	142	86	[12]
*Lactobacillus fermentum*	65	6.5	Mn^2+ ^(1.0), Co^2+ ^(2.0)	29.9	24.3	[38]
*Lactobacillus plantarum *NC8	60	7.5	Mn^2+ ^(1.0), Co^2+ ^(0.5)	43.42	40	[21]
*Lactobacillus sakei *23 K	30-40	5.0-7.0	Mg^2+ ^(0.8), Mn^2+ ^(0.8)	31.6	264	[24]
*Thermotoga maritima*	90	7.0	Mn^2+^(5.0), Co^2+^(1.0)	63	41.3	[26]
*Thermotoga neapolitana*	85	7.0-7.5	Mn^2+ ^(1.0), Co^2+ ^(1.0)	60	119	[10]
*Thermus *sp. IM6501	60	8.5	Mn^2+ ^(5.0)	NR	NR	[18]
*Bacillus stearothermophilus *IAM1101	65	6.0	MnP^2+P ^(1.0)	NR	NR	[39]

NR: Not Reported

Top: Optimal temperature

*: Substrate is L-arabinose

### Effects of temperature, pH and metallic ions on L-AI activity

*Shewanella *sp. ANA-3 L-AI was optimally active within a large range of temperatures from 15 to 35°C, and highly active at low temperatures preserving nearly 90% to 95% of its optimal activity at 4 to 10°C, respectively. At 40 to 45°C, the protein retained 90% to 85% of its activity, respectively, whereas at higher temperatures (> 45°C) the relative activity rapidly decreased. This pronounced activity at low temperatures distinguishes it from all previously described L-AIs (Table 1), and another interesting feature is that more than 85% of its maximal activity is maintained in a wide range of temperatures from 4 to 45°C. This behavior indeed promotes the use of this enzyme in agro-food industry. Study of the effect of pH on this L-AI demonstrates that this protein was highly active at pH 5.5-6.5. Furthermore, the enzyme retains more than 80% of its activity from pH 4.0 to 8.5. Remarkably, this protein preserves more than 60% of its relative activity at pH 3.0 and 3.5, whereas the majority of the earlier reported L-AIs have optimal pH values of 7.0-8.0 (Table 1). For instance, all L-AIs from *Bacillus *genera possess a rather narrow range of optimal pH from 7.0 to 8.5. Concerning L-AIs from lactic acid bacteria, the optimum pH values are more acidic spanning from 5.0 to 7.5, and the most acid tolerant candidate being *L. sakei *23 K L-AI functioned at pH 5.0-7.0 [24]. In contrast, some L-AIs act at highly alkaline conditions; such is the case of *Anoxybacillus flavithermus *L-AI that has an optimal pH range from 9.5 to 10.5 [25]. However, the acid tolerance is important mainly for industrial purposes. In fact, isomerization of D-galactose under acid pH prevents the formation of unwanted products [26,27].

**Figure 3:**
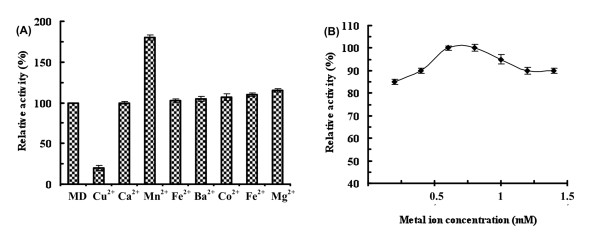
***Shewanella *sp. ANA-3 L-AI activity**. (A): Effect of divalent metal ions. (B): Effect of various concentrations of Mn^2+ ^(♦). "MD" indicates enzyme activity measured in a metal depleted solution. Activities at optimal metal ion concentrations were defined as 100%. Error bars represent the standard deviation from three independent experiments.

Treatment of the enzyme with ethylenediaminetetraacetic acid (EDTA) showed a 74% decrease in the activity. Amongst several metal ions only Mn^2+ ^restored the enzyme activity, which exceeded that observed for the metal-depleted enzyme by 1.6 fold (Figure 3A), at a concentration of only 0.6 mM Mn^2+ ^(Figure 3B). Analysis of the EDTA-treated enzyme by flame atomic absorption spectrometry demonstrates the presence of only manganese; thus highlighting its tight interaction with protein. This explains the residual activity (26%) obtained after enzyme EDTA treatment. Comparatively to all reported L-AIs, the present enzyme showed a weak requirement for metal ions for its optimal activity (Table 1). This feature is highly advantageous for its potential use in agro-food industry where many metal ions are not authorized, and therefore if used should be discarded from the final products, thereby increasing the process costs [7].

### *Shewanella *sp. ANA-3 thermal and pH stability studies

**Figure 4:**
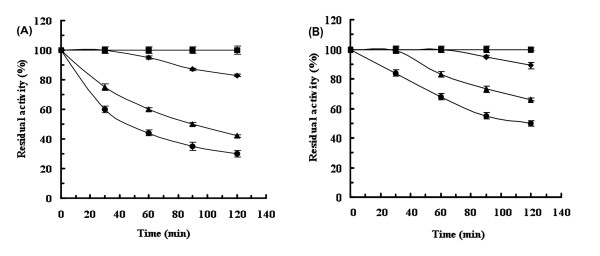
**Thermostability profiles of (A) the metal depleted wild-type *Shewanella *sp. ANA-3 L-AI and (B) in the presence of 0.6 mM Mn^2+^: (■) 35°C, (♦) 40°C, (▲) 45°C and (●) 50°C**. The initial activity was defined as 100%. Error bars represent the standard deviation from three independent experiments.

Enzyme stability is a crucial criterion for industrial applications. Our study showed that without addition of metal ions this enzyme was fully stable at temperatures below 35°C. At 40°C the enzyme upheld more than 80% of its activity after two hours, and at 45 and 50°C the enzyme stability decreased with half-lives of 90 and 40 min, respectively (Figure 4A).

After addition of 0.6 mM Mn^2+ ^the enzyme was completely stable for 1 hour at 40°C and preserved 90% of its activity after two hours of incubation. Addition of manganese clearly improved the thermal stability at 45 and 50°C, the enzyme retained more than 50% of its initial activity after two hours (Figure 4B). These results give evidence that Mn^2+ ^ions play a crucial role in the stabilization of the enzyme at high temperatures. This phenomenon is well reported for L-AIs, requiring metal ions for their stability (Table 1). Study of the enzyme stability at different pH values demonstrates that this biocatalyst has high half-lives in a wide pH range from 4.5 to 7.0 (Table 2). Furthermore, at pH 3.0 to 4.0 and 7.5 to 8.5 the half-lives dropped slightly. Variations of temperature from 4 to 30°C were not significant, but highlighting the stability of the *Shewanella *enzyme in a large range of temperatures and pH.

**Table 2:** pH-Stability of wild-type L-AI and its T268K mutant (values within brackets) at different temperatures without addition of metal ions.

			Temperature (°C)	
pH	4	10	20	30
			**Half-lives (*t*_1/2_, h)**	
3.0	27 ± 0.2 (39 ± 0.1)	28 ± 0.1 (38 ± 0.3)	27 ± 0.4 (38 ± 0.7)	27 ± 0.1 (38 ± 0.4)
3.5	32 ± 0.3 (39 ± 0.7)	33 ± 0.5 (39 ± 0.2)	32 ± 0.1 (39 ± 0.1)	31 ± 0.2 (40 ± 0.5)
4.0	35 ± 0.4 (40 ± 0.3)	34 ± 0.4 (41 ± 0.7)	35 ± 0.4 (41 ± 0.4)	36 ± 0.3 (41 ± 0.8)
4.5	39 ± 0.6 (42 ± 0.5)	39 ± 0.4 (43 ± 0.4)	38 ± 0.8 (43 ± 0.2)	39 ± 0.6 (42 ± 0.3)
5.0	41 ± 0.2 (42 ± 0.4)	42 ± 0.3 (43 ± 0.2)	40 ± 0.5 (44 ± 0.8)	41 ± 0.8 (42 ± 0.9)
5.5	43 ± 0.7 (43 ± 0.8)	44 ± 0.2 (44 ± 0.6)	44 ± 0.6 (44 ± 0.2)	43 ± 0.4 (43 ± 0.2)
6.0	43 ± 0.5 (43 ± 0.2)	44 ± 0.6 (44 ± 0.8)	44 ± 0.7 (44 ± 0.5)	43 ± 0.7 (43 ± 0.4)
6.5	43 ± 0.2 (44 ± 0.6)	44 ± 0.1 (44 ± 0.5)	44 ± 0.5 (44 ± 0.6)	43 ± 0.4 (44 ± 0.5)
7.0	44 ± 0.8 (44 ± 0.4)	44 ± 0.8 (44 ± 0.2)	44 ± 0.2 (44 ± 0.3)	43 ± 0.1 (44 ± 0.1)
7.5	43 ± 0.2 (43 ± 0.6)	43 ± 0.3 (43 ± 0.4)	43 ± 0.1 (43 ± 0.3)	43 ± 0.5 (43 ± 0.4)
8.0	41 ± 0.5 (41 ± 0.6)	41 ± 0.6 (42 ± 0.3)	42 ± 0.2 (42 ± 0.1)	42 ± 0.7 (42 ± 0.6)
8.5	40 ± 0.6 (40 ± 0.5)	40 ± 0.7 (40 ± 0.8)	40 ± 0.5 (40 ± 0.7)	40 ± 0.7 (40 ± 0.9)

Using sequence alignment, Lee et al. (2005) identified Lys269 in *Alicyclobacillus acidocaldarius *L-AI as an important determinant for enzyme's acid tolerance [15]. This hypothesis was confirmed by generating mutants that shifted the optimum pH by one unit towards more alkaline pH [15]. Guided by these studies, we have explored the influence of the non conserved lysine residue at position 268 on the biochemical properties of the *Shewanella *sp. ANA-3 enzyme (Figure 1). By site-directed mutagenesis we replaced the corresponding threonine by a lysine, and our data indicate that this mutation does not affect the enzymatic activity at different temperatures. As for the relative activity profile at different pH values, it seems that the T268K mutation increases the enzyme activity mostly at highly acidic pH. Remarkably, the wild-type *Shewanella *L-AI upholds more than 60% of its activity at pH 3.0 and the T268K mutant is even more acid-tolerant, retaining 80% of its activity at the same pH. Also, analysis of this mutant stability at different pH and different temperatures demonstrates that the T268 K L-AI mutant displays higher half-lives with low pH (3.0-4.5) when compared to the wild-type enzyme (Table 2). Similar half-lives were obtained for both mutant and wild-type *Shewanella *sp. ANA-3 L-AIs at pH's from 5.0 to 7.0. Remarkably, T268K L-AI mutant displays a maximal stability at low pH value of 5.5 and high value of 7.0. Thus, this mutant is not only more acid-tolerant than the wild-type enzyme, but also more stable under acidic pH and low temperatures. This takes more importance when we consider that an attractive enzyme, from industrial point of view, should be stable and efficient. Such data confirm the essential role of the K268 residue on both acid-tolerance and pH stability of L-AIs.

### Kinetic characterization and D-galactose bioconversion

Determination of the kinetic parameters of the *Shewanella *sp. ANA-3 L-AI showed that it has *K*_m _values of 33.7 and 52.1 mM for L-arabinose and D-galactose, respectively. Furthermore the *V*_max _was 164 mmole/s/mg for L-arabinose and 44 mmole/s/mg for D-galactose. To further explore the efficiency of this enzyme to produce D-tagatose, we carried out D-galactose bioconversion at different temperatures. As shown in Table 3 the highest bioconversion rate (34%) was obtained at 35°C. At lower temperatures including 30, 20, 10 and 4°C maximum bioconversion levels of 31%, 25%, 21% and 16% were obtained, respectively. These data underline the catalytic efficiency of this enzyme to produce D-tagatose at low temperature. In addition, analysis of Table 3 demonstrates that bioconversion rates are improved when temperature is increased, thus confirming previously reported results concerning the relationship between temperature and isomerization reaction equilibrium [13,15,16].

**Table 3:** Kinetics of D-galactose bioconversion rates using the purified enzyme (1 mg/ml) at different temperatures.

Temperature (°C)	4	10	20	30	35
**Time (h)**		**Bioconversion rate (%)**			
1.0	6.0 ± 0.1	6.0 ± 0.2	9.0 ± 0.1	10 ± 0.5	11 ± 0.1
2.0	9.0 ± 0.2	8.0 ± 0.5	12 ± 0.5	14 ± 0.2	16 ± 0.3
3.0	12 ± 0.1	12 ± 0.3	17 ± 0.8	19 ± 0.7	22 ± 0.4
4.0	15 ± 0.4	18 ± 0.7	20 ± 0.4	25 ± 0.9	28 ± 0.7
5.0	16 ± 0.8	20 ± 0.6	24 ± 0.6	29 ± 0.6	32 ± 0.6
6.0	16 ± 0.2	21 ± 0.2	25 ± 0.5	30 ± 0.4	34 ± 0.1
7.0	16 ± 0.3	21 ± 0.5	25 ± 0.2	31 ± 0.3	34 ± 0.8

Such enzyme features are very attractive from an industrial point of view. In fact, until now the majority of characterized L-AIs are thermoactive and not acid-tolerant. Generating a new acid-tolerant L-AI being highly active at low temperatures would allow the efficient bioconversion of D-galactose into D-tagatose in dairy products during fermentation and/or storage.

## Conclusions

The L-AI from psychrotolerant *Shewanella *sp. ANA-3 is highly active at a wide pH range and converts efficiently D-galactose into D-tagatose even at low temperatures. These biochemical properties are of interest for industrial purposes. Indeed, isomerization of D-galactose at acid pH prevents the formation of unwanted side-products and high stability at low temperatures is convenient for food industry applications. The T268K mutation did not change enzyme behavior regarding temperature but clearly enhanced the enzyme activity and stability at low pH values.

Further work will target the overexpression of this particular enzyme in a food-grade system such as lactic acid bacteria. Given the attractive properties of the enzyme it would be of high interest to employ it as a model for optimization of the bioconversion of D-galactose into D-tagatose in milk and dairy products such as yoghurts. In order to improve the suitability of this biocatalyst for industrial applications it is important to increase its affinity to D-galactose. For this purpose crystallization followed by the determination of the three-dimensional structure of this protein in complex with substrates by X-ray crystallography in combination with protein engineering are underway. Such data will allow improving the efficiency of other food-grade L-AIs.

## Methods

### Bacterial strains, plasmids and media

*Shewanella *sp. ANA-3 strain was routinely grown as reported by Saltikov et al. [28]. *Escherichia coli *BL21 (DE3) were used in this study as host strains. Culture of different *E. coli *strains was done in Luria Bertani (LB) medium. These media were supplemented, when necessary, with ampicillin (100 μg/ml) and IPTG (Isopropyl β-D-thiogalactopyranoside) at 160 μg/ml. The pGEMT-Easy (Promega) and pET-15b plasmids (Novagen) were used according to the manufacturer's instructions.

### DNA manipulation and PCR

Genomic DNA was prepared as reported by Johnson [29]. Preparation of plasmid DNA, digestion with restriction endonucleases and separation of fragments by agarose gel electrophoresis were performed as described by Sambrook et al. [30]. PCRs were carried out using Gene Amp^® ^PCR System 9700 (Applied Biosystems). The amplification reaction mixtures (100 μl) contained High Fidelity Taq amplification buffer, 20 pmol of each primer, 100 ng of DNA template, and 10 units of High-Fidelity Taq DNA polymerase (Invitrogen). The cycling parameters were 94°C for 5 min, followed by 40 cycles of 94°C for 30 s, 55°C for 60 s, and 72°C for 120 s. The amplified fragment containing the *Shewanella *sp. ANA-3 *araA *gene was sequenced using an automated DNA sequencer (Applied Biosystems).

### Cloning and over-expression of the *araA Shewanella *sp. ANA-3 gene

Aiming the amplification of the *Shewanella *sp. ANA-3 *araA *gene, we designed two primers based on the complete genome available in the NCBI databank. Oligonucleotide sequences were F-araA ^5'^GATAAGCACTATTTGCGTAAGCATG^3' ^and R-araA ^5' ^TTATTGCTGCGCAGAATTATAAACCTCTTGCTAAACCG^3'^. Chromosomal DNA isolated from *Shewanella *sp. ANA-3 strain was used as a template and High-Fidelity Taq DNA polymerase for amplification. The PCR product was purified using QIAquick Gel Extraction Kit (QIAGEN^®^) by following the manufacturer's instructions. Subsequently, the resulting fragments were cloned in pGEMT-Easy Vector (Promega) and transformed into *E. coli *BL21 (Invitrogen) competent cells. Recombinant white clones were selected on LB agar medium added with ampicillin (100 μg/ml), IPTG (160 μg/ml) and X-gal (100 μg/ml). Then, the *araA *gene was subcloned in the pET-15b (Novagen) under the control of the T7 promoter with six histidines at the N-terminal end of the protein. The T268K L-AI mutant was generated by using a site-directed mutagenesis Kit purchased from Stratagene (used as recommendation by supplier) and oligonucleotides T268Kd ^5'^AACCTCACTGGCATGAAAGGATTACCCGGACTG^3' ^and T268Kr ^5' ^CAGTCCGGGTAATCCTTTCATGCCAGTGAGGTT ^3'^.

### Amino acid sequence alignment

The multiple alignment of the L-AI amino-acid sequence was done using the program ClustalW [31] and the figure rendering was done using the ESPript sequence analysis server [32].

### Crude cell-lysate preparation and enzyme purification

The recombinant *Escherichia coli *strains, overexpressing *Shewanella *sp. ANA-3 L-AI, were grown in LB medium with ampicillin (100 μg/ml). From this culture a second one was launched under the same conditions at 200 rpm and 37°C. The culture was supplemented with IPTG (0.5 mM) at OD_600 nm _of 1.0 and subsequently incubated at 37°C and 220 rpm for over-night. Cells were harvested by centrifugation (6500 × *g *for 10 min at 4°C) and the pellets were re-suspended in 100 mM MES buffer at pH 5.5 supplemented with one Complete™ protease inhibitor cocktail tablet (Roche^®^). Cell disruption was carried out, twice, using a French Press at 1000 psi. For the purification, crude cell extract from the *E. coli *BL21 (DE3) strain was centrifuged (30 000 × *g*, for 30 min at 4°C). Proteins precipitated between 60 and 70% ammonium sulphate saturation, suspended in 100 mM MES buffer pH 5.5, concentrated and desalted in centrifugal micro-concentrators (Amicon, Inc) with a 30 kDa cut-off membrane. Then, an affinity chromatography was done using IMAC column on the ÄKTA Purifier FPLC system (Amersham Pharmacia Biotech) equilibrated with 100 mM MES buffer pH 5.5. Proteins were eluted at a flow rate of 3 ml/min using a linear imidazole gradient ranging from 0 to 500 mM in the same MES buffer with imidazole (500 mM). Fractions containing L-AI activity were pooled and the final purification phase was performed using size exclusion chromatography on a Sephacryl S-200 column (GE-Healthcare).

### Protein quantification, electrophoresis and molecular mass determination

Protein concentration was determined using Bradford's method with bovine serum albumin as standard [33]. The protein samples were separated in 12% SDS-PAGE gel according to the Laemmli method, and bands were visualized by Coomassie brilliant blue R-250 (Biorad) staining. The estimated molecular mass of the purified L-AI was determined by size exclusion chromatography.

### Enzyme activity assays

L-AI activity was measured by determining the amount of formed L-ribulose or D-tagatose. Under standard conditions, the reaction mixture contained 100 μl of enzyme (L-AI) preparation at a 1 mg/ml, 50 mM of L-arabinose (D-galactose) and 100 mM MES buffer (pH 5.5) in a final volume of 1 ml. The reaction was stopped by heating the mixture at 99°C for 2 min. The amount of L-ribulose (or D-tagatose) was colorimetrically determined using the cystein-carbazol-sulphuric-acid method and the absorbance was measured at 560 nm [34]. D-Tagatose production was also detected with high-performance ionic chromatography (HPLC) using a Polypore CA column (250 × 4.6 mm). The products were separated by isocratic elution with water at a flow rate of 0.3 ml/min and detected with a refractive index detector (SHIMADZU, Refractive Index Detector). Solutions of 5 g/l D-galactose and 5 g/l D-tagatose were used as standards.

One unit of L-AI activity was defined as the amount of enzyme catalyzing the formation of 1 μmol keto-sugar per min under the above-specified conditions.

### Temperature, pH and thermostability profiles

The effect of temperature on the activity was studied by incubating the purified protein at temperatures from 4 to 55°C. The enzyme pH profile was obtained at pH values between 3.0 and 8.5 (3.0 to 5.0 with sodium acetate buffer, 5.5 to 7.0 with MES buffer and 7.5 to 9.0 with Bicine buffer). Determination of the enzyme stability as a function of temperature and pH was carried out by measuring the residual activity at periodic intervals.

### Effect of metal ions on the enzymatic activity

*Shewanella *sp. ANA-3 L-AI was purified as described in the enzyme purification section but without addition of metal ions to the crude extract and dialyzed against 100 mM MES buffer (pH 5.5) containing 10 mM EDTA for 48 h at 4°C (buffer renewed twice). Subsequently, we twice dialyzed the EDTA-treated enzyme against 100 mM MES buffer (pH 5.5). Thereafter, the enzyme was pre-incubated during 10 min in solutions containing metal ions (CuSO_4_, CaSO_4_, MnSO_4_, FeSO_4_, BaSO_4_, CoSO_4_, FeSO_4_, MgSO_4_) followed by L-AI activity assays under standard conditions. The optimal metal ion concentrations were monitored by incubating the enzyme samples at various concentrations followed by activity assays.

### Flame atomic absorption spectrometry analysis

For sample preparation, each mineralized sample was employed at a final concentration evaluated to 10 mg/ml. A Perkin-Elmer Analyst 200 atomic absorption (Norwalk, USA), equipped with a deuterium lamp background correction system, was used for metal binding quantification. Hollow cathode lamps (Perkin-Elmer) were used as primary radiation source. Analytical measurements were based on time average absorbance, under conditions recommended by the manufacturer.

### Determination of kinetic parameters

Kinetic parameters were determined on the basis of Lineweaver-Burk plots. Assays were carried out in 100 mM MES buffer (pH 5.5), 0.6 mM Mn^2+ ^and 1 to 800 mM substrate (L-arabinose or D-galactose). Samples were incubated at 35°C and the amount of keto-sugar generated (L-ribulose or D-tagatose) was determined by the cysteine-carbazole-sulfuric acid method.

### Bioconversion assays

Bioconversion of D-galactose into D-tagatose was monitored in a solution containing 100 mM MES buffer (pH 5.5), 0.6 mM Mn^2+ ^and 50 mM D-galactose. Kinetic conversion of D-galactose was investigated for 7 h at 4, 10, 20, 30 and 35°C. Samples were taken periodically and the concentration of generated D-tagatose was determined as described earlier and confirmed by HPLC as indicated in the enzyme assay section.

## List of abbreviations

L-AI: L-arabinose isomerase; MES: 2(-N-morpholino)ethanesulfonic acid; EDTA: Ethylenediaminetetraacetic acid; HPLC: High-performance liquid chromatography; IPTG: Isopropyl β-D-thiogalactopyranoside; PCR: Polymerase Chain Reaction; RT: Retention Time; SDS-PAGE: Sodium Dodecyl Sulfate Polyacrylamide Gel Electrophoresis.

## Competing interests

The authors declare that they have no competing interests.

## Authors' contributions

MR participated in the design of the study, experiments analysis and writing the manuscript. GB participated in the molecular biology experiments. IR participated in the biochemical experiments. SB participates in the construction of mutant and expression of proteins. EM participated in the design of the work. RH participated in the conception of mutant. AN participated in the design of the work and helped in drafting the manuscript. All authors read and approved the final manuscript.
